# Effect of Structured Educational Program on Practices of Radiation Safety Measures Among Health Care Providers in Urology Operation Theater

**DOI:** 10.7759/cureus.15765

**Published:** 2021-06-20

**Authors:** Vijay Kumar, Atanu Kumar Pal, Sreerag Ks, Ramanitharan Manikandan, Lalgudi N Dorairajan, Sidhartha Kalra, Saravanan Kandasamy, Mujahid Khan

**Affiliations:** 1 Urology and Renal Transplantation, Jawaharlal Institute of Postgraduate Medical Education and Research, Pondicherry, IND; 2 Urology and Renal Transplantation, Jawaharlal Institute of Post Graduate Medical Education and Research, Pondicherry, IND; 3 Medical Physics, Jawaharlal Institute of Postgraduate Medical Education and Research, Pondicherry, IND

**Keywords:** structured education program, health care providers, radiation, urology operation theater, daily practice

## Abstract

Introduction

Endourologists are at increased risk of exposure to radiations. Many studies are available that have studied awareness in doctors in general, but very few studies available regarding any intervention to improve the knowledge of radiation safety measures. We have made an attempt to study the role of an educational intervention to improve the knowledge of our Jawaharlal Institute of Postgraduate Medical Education and Research (JIPMER) urology operation theater health care providers (HCPs).

Materials and methods

Our study was an Interventional study (prospective clinical trial), conducted in the Department of Urology, JIPMER from January 2017 to March 2018. All, that is, 40 operation theater HCPs were given a questionnaire as baseline. The baseline response was compared to the response after the Structured Education Program (SEP) by using the same questionnaire. The knowledge of participants before SEP was compared with the knowledge after SEP using the chi-square test. All statistical analysis was carried out at a 5% level of significance and p-value < 0.05 was considered as significant.

Result

In our study after SEP, participants use of lead apron has increased from 72.5% to 92.5%, indicating improvement. There is an increase in the use of thyroid shield from 22.5% to 95%. In our study after SEP, knowledge about background radiations improved in participants from 25% to 87.5%. Knowledge about Radiation dose of chest X-ray improved from 22.5% to 52.5%. Knowledge about ALARA (As Low As Reasonably Achievable) improved from 47.5% to 95% after SEP. Knowledge that MRI and USG do not have ionizing radiation improved from 62.5% to 97.5%, and from 75% to 92.5% for MRI and USG, respectively, after SEP. Regarding organ sensitivity, 100% HCPs had given correct answers after SEP as compared to 80 before SEP.

Conclusion

Our study shows that SEP at regular intervals has made significant improvements in daily practice in operation theater HCPs. SEP has increased the use of radiation protective gears among HCP. Hence we recommend SEP at regular intervals for urology operation theater HCPs for a healthy and safe working environment.

## Introduction

In Urologic practice, Endourology is day to day expanding branch and fluoroscopy in the form of C-arm is used in almost all the cases of Endourology. So, Endourologists are at increased risk of exposure to radiations [1]. Urologists have a key role in controlling the radiation exposure to themselves and other medical and paramedical staff around [2]. Although these radiation exposures are minimal, the principle of ALARA (As Low As Reasonably Achievable) should be followed. Depending on the dose and duration of exposure to radiation, radiations are always having negative biological effects on living beings [3]. Despite so many studies, no threshold dose for stochastic effects of radiation is defined that can cause cancer [4]. The maximum yearly whole-body exposure recommended by the International Commission on Radiation Protection (ICRP) is 20 mSv per year, averaged over defined periods of five years [5]. There are no standardized protocols available regarding training to improve the knowledge of health care professionals (HCPs) and may be lacking [2].

Radiation exposure is present in many departments, including orthopedics, radiodiagnosis, cardiology and radiation oncology departments [6]. Many studies are available that have studied awareness in doctors in general, but only a few studies are available in the literature regarding awareness of radiation exposure among urologists [7-10] and similarly very few studies available regarding any intervention to improve the daily practice of radiation safety measures. We have made an attempt to study the role of an educational intervention to improve the knowledge of our Jawaharlal Institute of Postgraduate Medical Education and Research (JIPMER) urology operation theater HCPs. As such no study is available that has studied this aspect of the problem.

## Materials and methods

Our study was an Interventional study (prospective clinical trial), conducted in the Department of Urology JIPMER (Jawaharlal Institute of Postgraduate Medical Education and Research) from January 2017 to March 2018. The approval of the Post Graduate Medical Research Committee (PGRMC) and Institute Ethics Committee (Humans Studies) were obtained for study. Informed consent was taken from all participants. All HCPs participated in the study, according to the following criteria. Inclusion criteria: All (40) operation theater HCPs (including Urology and Anaesthesia Faculty members, Urology and Anesthesiology Senior Residents, Surgery Junior Residents posted in Urology, Nurses and OT Technician in both Urology Major and Minor OTs) in urology OT who are working or likely to work for at least one month.

Exclusion criteria

Operation theater HCPs not giving consent for participation in the study. All operation theater HCPs were given a questionnaire and a checklist was used to assess their level of practice (Appendix). All questionnaires were administered and collected by the urology office clerk and it was made sure that no participant is using any kind of help while filling the questionnaires. A Structured Education Program (SEP) in the form of lectures and demonstrations was developed and validated. The data on the level of knowledge was assessed by using a structured questionnaire. At the beginning of the study, all operation theater HCPs were assessed for their baseline knowledge on radiation safety measures by a questionnaire. This was done in the first four weeks of study to ensure every participant to be covered. After this, a SEP was provided to operation theater HCPs once every month for eight months, so that to ensure that every participant got two SEPs and intervention remained the same for each participant. In the last seven months at the end of education program, a same questionnaire and radiation safety checklist were used for HCPs. Compliance with radiation safety was assessed twice weekly, every month after the intervention (SEP), after which results were analyzed.

The distribution of the categorical variables such as age, gender, education status, etc. of an HCP was expressed as frequency and percentages. The continuous data on age, gender, occupational status, level of knowledge were expressed as frequencies and percentages. Knowledge of participants before SEP was compared with knowledge after SEP using chi-square test. The level of knowledge was further categorized into knowledge of age groups, gender groups and occupational groups and the association categorical variables were carried out using the chi-square test. All statistical analysis was carried out at a 5% level of significance and p-value < 0.05 was considered as significant.

## Results

The study was conducted from January 2017 to March 2018 in JIPMER, Pondicherry. A total of 40 Urology operation theater HCPs were assessed before and after the structured education program. Sociodemographic distribution of study participants is given in Table 1.

**Table 1:** Sociodemographic distribution of study participants.

Age groups (in years)	Frequency (n)	Proportion (%)
20-35	29	72.5%
36-50	11	27.5%
Gender		
Male	12	30%
Female	28	70%
Job status		
Nurses	16	40%
Urotechnicians	03	7.5%
Non-urologists doctors	07	17.5%
Urologists	14	35%

Comparison of practice of radiation protective gears in participants before and after the SEP is reported in Table 2. In our study after SEP, participants use of lead apron has increased from 29 (72.5%) to 37 (92.5%), indicating improvement. There is an increase in the use of thyroid shield from 9 (22.5%) to 38 (95%).

**Table 2:** Comparison of Practice of radiation protection measures among urology operation theater health care providers before and after Structured Educational Program (SEP).

Use of radiation protection measures	Correct answers before SEP	Correct answers after SEP	p-value
Lead apron	29 (72.5%)	37 (92.5%)	<0.0185
Thyroid shield	9 (22.5%)	38 (95%)	<0.001
Dosimeter	5 (12%)	13 (32.5%)	<0.0322

Participants who answered correctly the questions on knowledge before and after the SEP are reported in Table 3. In our study after SEP, knowledge about background radiations improved in participants from 10 (25%) to 35 (87.5%). Knowledge about Radiation dose of chest X-ray improved from 9 (22.5%) to 21 (52.5%). Knowledge about ALARA (As Low As Reasonably Achievable) improved from 19 (47.5%) to 38 (95%) after SEP. Knowledge that MRI (magnetic resonance imaging) and USG (ultrasound sonography test) did not have ionizing radiation improved from 25 (62.5%) to 39 (97.5%), and from 30 (75%) to 37 (92.5%) for MRI and USG, respectively, after SEP. Regarding organ sensitivity 100 % HCPs had given correct answers after SEP as compared to 80 before SEP.

**Table 3:** Comparison of knowledge of operation theater HCPs before and after Structured Educational Program (SEP). ALARA: As Low As Reasonably Achievable; CT: computerized tomography; MRI: magnetic resonance imaging; USG: ultrasound sonography; IVU: intravenous urography; HCPs: health care providers.

Questions	Correct answers before SEP	Correct answers after SEP	p-value
Background radiations	10 (25%)	35 (87.5%)	<0.001
Radiation dose of chest X-ray	9 (22.5%)	21 (52.5%)	0.005
Meaning of ALARA	19 (47.5%)	38 (95%)	<0.001
Radiation sensitivity of organ (Gonads)	32 (80%)	40 (100%)	NA
Radiation absorption from X-ray abdomen	24 (60%)	39 (97.5%)	<0.001
Radiation absorption from CT	9 (22.5%)	38 (95%)	<0.001
Radiation absorption from MRI	25 (62.5%)	39 (97.5%)	<0.001
Radiation absorption from USG	30 (75%)	37 (92.5%)	0.033
Radiation absorption from IVU	13 (32.5%)	33 (82.5%)	<0.001

Among seven non-urologist doctors, only two (28.6%) were using a thyroid shield before SEP, but after SEP all (100%) started using thyroid shield. Already five (71.4%) were using the lead apron before SEP, which increased to 6 (85.7%). Nobody was using the dosimeter before but after SEP two started using dosimeter. 

Among 19 nurses who participated in the study use of lead apron, thyroid shield and dosimeter increased after SEP from 14 (73.7%) to 18 (94.7%) (p = 0.075), 5 (26.3%) to 18 (94.7%) (p < 0.001) and 3 (15.8%) to 7 (36.8%) (p = 0.140), respectively. 

Among 14 urologists who participated in study improvement in the use of lead apron, thyroid shield and dosimeter after SEP was 10 (71.4%) to 13 (92.9%) (p = 0.138), 2 (14.3%) to 13 (92.6%) (p < 0.001) and 2 (14.3%) to 4 (28.6%) (p = 0.356), respectively.

## Discussion

Radiation safety is a concern for patients, physicians, and staff in many departments. According to a cross-sectional survey of HCPs in Trinidad 85 out of 118 HCPs had no formal training regarding safe practices and those who had formal training performed better than those individuals without training [11]. In another study among pediatric anesthesiologists it has been shown that despite universal exposure to ionizing radiation from X-rays, they do not routinely adhere to strategies designed to limit the intensity of this exposure and rarely work in institutions in which a culture of radiation safety exists. They highlight the need to improve radiation safety education, the need to change the safety culture within the operating rooms and imaging suites [12]. Elkoushy et al. reported that compliance in the use of thyroid shields, dosimeters, and lead-impregnated glasses and gloves could be improved among endourologists [13]. A well-developed radiation safety climate fosters positive radiation safety behaviors, which may partially be explained through improved radiation safety knowledge transfer [14].

In our study after SEP, participants use of lead apron has increased from 29 (72.5%) to 37 (92.5%), (p = 0.0185) indicating significant improvement. There is a significant increase in the use of thyroid shield from 9 (22.5%) to 38 (95%) (p < 0.001). There was a minimal increase in the use of dosimeter from 5 (12%) to 13 (32.5%), but it was also statistically significant (p = 0.032). The difficulty in getting the dosimeter could be the reason for the not expected rise in use of dosimeter among the HCP. Overall SEP worked well and showed results in the form of increased usage of radiation protective gears. Similarly, Freidman et al. [2] showed that trainees that were educated about the radiation safety in urology department were having more compliance toward protective equipment, dosimeter, and principles of ALARA.

Soye and Paterson [15] reported that one-third of doctors who were trained in radiation safety programs were more near to correct answers than the doctors who never underwent training.

In our study after SEP knowledge about background radiations improved in participants from 10 (25%) to 35 (87.5%) (p < 0.001), knowledge about Radiation dose of chest X-ray improved from 9 (22.5%) to 21 (52.5%) (p = 0.005) and knowledge about ALARA improved from 19 (47.5%) to 38 (95%) (p < 0.001). After SEP knowledge that MRI and USG did not have ionizing radiation improved from 25 (62.5%) to 39 (97.5%), (p < 0.001) and from 30 (75%) to 37 (92.5%) (p = 0.033) for MRI and USG, respectively. Regarding organ sensitivity approximately 100% HCPs had given correct answers. Significant impact of SEP seen in improvement in knowledge of urology operation theater HCPs about ionizing radiation in the medical area as shown in below given studies.

Indeed, previous studies indicated that formal education of radiation safety measures of physicians has contributed to greater awareness for radiation dosages and risks [15]. Even brief exposure of education programs improves the knowledge of ALARA principles and its implementation [16]. Food and Drug Administration recommends that each physician doing fluoroscopy guided procedures should receive an education, so that he can assess the risks, benefits and limit exposure on a case to case basis [17].

Freidman et al. [2] showed that 50 trainees who had education program were having more compliance with protective equipment, dosimeters, and principles of ALARA and greater knowledge about recommended exposure limits. Lancaster et al. [18] found a significant correlation between shorter fluoroscopy time and SMART (Safety, Minimization, and Awareness Radiation Training) exposure. In their study multivariate regression analysis revealed that fluoroscopy time was significantly shorter with SMART. It was 45 seconds with SMART and 102 seconds without SMART. Chun-sing et al. [19] in his study found that a majority of subjects (80%) want to know about radiation safety and awareness on the job training. This is of utmost importance as day by day we are having increased exposure to procedures in which there is an increased exposure to radiations. It has been already considered to introduce intensive training courses on theoretical aspects of radiation exposure for junior doctors and implementing training sessions on radiation protection in our medical schools [20]. Horowitz et al [21] in their study showed that after the lecture on radiation safety measures, there was a fall in prescribing CT scan and there was an increase in prescribing alternative imaging in the form of MRI and USG.

According to checklist, there is a 99.9% increase in usage following radiation safety measures. But we have observed that after a time gap of a few months, usage of radiation safety measures again deteriorated. So in view of the significant impact of education program and deterioration after a time gap, we stress for the regular structured education program at regular intervals to strengthens the daily practice of operation theater HCPs.

A recent study has shown that a brief brochure of information about CT scan improves the understanding. Our study is not powered to recommend which type of education program is more impacting in improving the daily practice of physicians.

In our study, many factors seem to be contributing to the poor knowledge of our study participants before the education program. Participants were never undergone formal training on this topic. Inadequate and restricted availability of radiation safety equipment that is dosimeters, eye glasses and leaded gloves and thyroid shields.

The limitations of our study, it is a small population study. Further large population studies needed to have greater insight. In our study question was asked regarding taking up education program in the past, but not specified as what is a source of education, whether it was in the form of mandatory internet training or training through the department of urology or self gained knowledge after reading articles, which is done in the study by Freidman et al. [2] and it showed that the most common education source on radiation safety was mandatory graduate medical education training (55%). Audiovisual aids could have been used for a better understanding of the program by the HCPs.

In view of the above discussion, we recommend a regular interval structured education program for improvement of usage of radiation safety measures in all urology HCPs. Radiation protective gears, making companies should also take necessary action to improve the ergonomics of the protective gears.

## Conclusions

The level of knowledge of radiation safety measures among urology operation theater HCPs was low. In keeping with the ALARA principle, minimum radiation exposure of the medical staff is mandatory; however, this is not always put into daily endourology practice. Our study shows that SEP at regular intervals has made significant improvements in daily practice in operation theater HCPs. SEP has increased the use of radiation protective gears among HCP. Hence we recommend SEP at regular intervals for urology operation theater HCPs for a healthy and safe working environment.

## Appendices

Questionnaire for Health Care Providers

1. Did you take any radiation safety training program?

Yes

No.

2. If you did not take a radiation safety program; do you wish to get it?

Yes.

No.

3. Have you ever read a medical article about radiation safety?

Yes.

No.

4. How often do you expose to radiation during endourological procedures?

More than 3 times per week.

1-3 times per week.

Less than 1 time per week.

I don’t expose to radiations.

5. How often do you use radiation protection clothes during fluoroscopy-guided endourological procedures?

6. How far from fluoroscope do you stand without any protection?

1 meter.

2 meter.

5 meter.

7. What do you think about practical use of protective clothes listed here?

8. How much radiation, in millisieverts (mSv), is a person exposed to, on average, every year, from natural background radiation?

0.24.

2.4

24

240

No idea

9. What is the approximate radiation dose, in mSv, of a Chest X-ray?

0.02

0.2

2

20

No idea.

10. Which of following explains the ALARA principle?

As Low As Reasonably Achievable.

Allowable Administered Radiation.

Assurance Limits Applied To Radiation.

No idea.

11. Please score the following organs in order of radiation sensitivity.

12. If a chest x-ray counted as 1 unit (u), how many units would a patient absorb in the following investigations?

**Table 4:** Response for question 5 (How often do you use radiation protection clothes during fluoroscopy-guided endourological procedures?) of Questionnaire for Health Care Providers.

	Never	Sometimes	Generally	Always	No idea
Lead apron					
Thyroid shield					
Dosimeter					

**Table 5:** Response for question 7 (What do you think about practical use of protective clothes listed here?) of Questionnaire for Health Care Providers.

	Very good	Good	Bad	Very bad	No idea
Lead apron					
Thyroid shield					
Leaded gloves					
Eye glasses					

**Table 6:** Response for question 11 (Please score the following organs in order of radiation sensitivity) of Questionnaire for Health Care Providers.

	Insensitive	Sensitive	Very sensitive	No idea
Bladder				
Gonads				
Kidney				

**Table 7:** Response for question 12 (If a chest x-ray counted as 1 unit (u), how many units would a patient absorb in the following investigations?) of Questionnaire for Health Care Providers. CT: computed tomography; MRI: magnetic resonance imaging; USG: ultrasound sonography test.

	0	1-10x	10-50x	50-100x	>300x
Abdomen X-ray					
Abdominal CT					
Abdominal MRI					
Abdominal USG					
Intravenous urography					
